# Reminiscences of Samuel Latham Mitchill, M.D., LL.D.

**Published:** 1859-11

**Authors:** John W. Francis


REMINISCENCES OF SAMUEL LATHAM MITCHILL, M.D., LL.D. By *John W. Francis*, M.D. etc., 1859. The friends and admirers of Dr. Mitchill may consider themselves as exceedingly fortunate in having so able and distinguished a biographer as Dr. Francis, whose glowing and eloquent pen has so forcibly and so beautifully portrayed the characteristic traits of that illustrious philosopher. There is no man in the country who understood Dr. Mitchill so thoroughly as Dr. Francis; for many years they were on terms of great intimacy, and in almost daily communication, discoursing now upon medicine, now upon some branch of natural science, now upon government or politics, and anon upon the fine arts, philosophy, or divinity; like twin brothers, born not for each other merely, nor for their own generation only, but for all time. It was fit then that the survivor should render this homage to his deceased comrade; and no one can read these reminiscences without being impressed with the conviction that he has performed his task most worthily alike of himself and of his friend.

Dr. Mitchill was born in Queen's County, Long Island, New York, on the 20th of August, 1764, his father being a farmer, and a member of the Society of Friends. He received his medical degree in the University of Edinburgh, at a time when that institution was adorned by the talents and eloquence of a Cullen, a Black, a Duncan, and a Monro. Among his companions there were Casper Wistar, James Mackintosh, Richard S. Kissam, and Thomas Addis Emmet, worthy compeers of such a youth. Upon his return to America, he studied law, natural history, chemistry, mineralogy, geology, and ethnology, evidently thinking, like Adam Clark, that it was impossible for him to have too many irons in the fire. Not long afterwards he was appointed Professor of Agriculture and Chemistry in Columbia College, and of Natural History, Botany, and Materia Medica in the College of Physicians and Surgeons of New York. He aided in founding the Military Academy at West Point, established with Miller and Smith the New York Medical Repository, carried on a large correspondence with scientific men in various parts of the world, contributed numerous papers to different periodicals both at home and abroad, and performed an immense number of experiments, illustrative of various chemical, medical, and philosophical subjects. Besides all this immense labor, he was constantly engaged in benevolent enterprises. Prof. Mitchill was a man not only of great learning and research, but of an enlarged philanthropy, losing no opportunity of doing good. He had a strong perception of the beautiful in art as well as in nature; and often gave vent to his emotions in verse, replete with genuine taste and poetic imagery. His memory was wonderful, or, in the language of his admirable biographer, herculean. His personality may be gathered from the following paragraph:—

"In the prime of his manhood Dr. Mitchill was about five feet ten inches in height, of a comely, rather slender and erect form; in after life he grew more muscular and corpulent, and lost somewhat of that activity which characterized his earlier days. He possessed an intelligent expression of countenance, an acquiline nose, a gray eye, and full features. His dress at the period he entered into public life was after the fashion of the day, the costume of the times of the Napoleonic consulate; blue coat, buff-colored vest, smalls, and shoes with buckles. He was less attentive to style of dress in his maturer years, and abandoned powder and his cue. From a hemorrhagic tendency of his chest at the age of seventeen years, he adopted exercise on horseback, and was fortunate enough to avert the progress of pulmonary evils. His personality, however, varied in advanced life with the cogitations of his graver years, and he might at times be seen without hat or overcoat, exposed to the vicissitudes of inclement weather. His robustness preserved his full features, and to the last not a wrinkle ever marked his face, nor did lapse of years modify his thirst for knowledge, or his cordial and prompt and sprightly utterance; thus setting at naught the declaration of the poet,—

> 'Old age doth give by too long space,
> Our souls as many wrinkles as our face.'"

Dr. Mitchill died in September, 1831, in the sixty-seventh year of his age. His remains repose in Greenwood Cemetery, where a beautiful monument was erected to his memory by his still surviving widow. He never had any children.

